# Neuroanatomical Changes Underlying Vertical HIV Infection in Adolescents

**DOI:** 10.3389/fimmu.2019.00814

**Published:** 2019-04-17

**Authors:** Xiao Yu, Lei Gao, Haha Wang, Zhuang Yin, Jian Fang, Jing Chen, Qiang Li, Haibo Xu, Xien Gui

**Affiliations:** ^1^Department of Radiology, Zhongnan Hospital of Wuhan University, Wuhan University, Wuhan, China; ^2^Publicity Department, Zhongnan Hospital of Wuhan University, Wuhan University, Wuhan, China; ^3^Training Centre of AIDS Prevention and Cure of Hubei Province, Zhongnan Hospital of Wuhan University, Wuhan University, Wuhan, China

**Keywords:** AIDS, brain development, structural MRI, HIV exposure, children, mother-to-child transmission

## Abstract

**Purpose:** The aim of this study was to investigate how human immunodeficiency virus (HIV) affects brain development in adolescents, what are susceptible brain regions, and how these brain structural changes correlate with cognitive abilities.

**Methods:** We used structural magnetic resonance imaging to examine gray matter volume and cortical thickness in 16 HIV-infected children (mean age = 13.63 years) and 25 HIV-exposed uninfected children (mean age = 13.32 years), 12 of them were subjected to a 1-year repetitive magnetic resonance scan of the brain. Five neurocognitive tests were performed on each subject to assess cognitive performance in different areas.

**Results:** Cross-sectional studies showed that the gray matter volume of HIV-infected children was widely reduced (mainly in the bilateral frontal, temporal, and insular regions, and cerebellum). The changes in cortical thickness were mainly due to thinning of the right temporal lobe and thickening of the left occipital lobe. Longitudinal studies showed that the gray matter volume reduction of HIV-infected children after 1 year mainly occurs in the advanced functional area of the right prefrontal, parietal lobe and the motor area, cortical thinning of brain regions were sensorimotor cortex and the limbic system. The gray matter volume of the bilateral cerebellum was positively correlated with the performance of the Wisconsin Card Sorting Test, while the cortical thickness of the right dorsolateral prefrontal cortex was negatively correlated with this test.

**Conclusion:** This study found that HIV-infected pubertal children showed a delayed cortical maturation with atrophy. This abnormal pattern of cortical development may be the structural basis for cognitive impairment in HIV-infected children.

## Introduction

Acquired immune deficiency syndrome (AIDS)-related encephalopathy is one of the most serious complications of AIDS and is more common in children than in adults (1). At present, there are nearly 2 million children living with human immunodeficiency virus (HIV) in the world, almost all newly infected children are infected through mother-to-child transmission (2). Effective antiretroviral therapy (ART) can significantly reduce mortality and the incidence of HIV encephalopathy (3). Although the children's HIV/AIDS has been transformed into chronic, controllable disease patterns, due to the HIV virus, antiretroviral drugs and a variety of environmental factors, a series of neuropsychological defects can still appear on HIV-infected (HIV+) children, sometimes even after the treatment has fully been accepted (4).

Currently, some studies have reported and summarized the neurocognitive impairment and behavioral abnormality in children infected with HIV. For example, language barriers, delayed motor development, poor school performance, etc., and some may even have psychological problems such as anxiety and depression (5, 6). Although highly active antiretroviral therapy can improve the cognitive function of HIV+ children in the long run (7), there are also studies showing that cognitive impairment will exist for a long time (8). It will affect their future learning, work performance and social practice, we need to focus on long-term outcomes of nervous system development to inform treatment options and guide early intervention treatment strategy to prevent deterioration of cognitive function.

The rapid development of neuroimaging has provided us with a powerful tool for studying brain development in children. Current structural magnetic resonance imaging (MRI) studies have observed some changes in brain structure in HIV+ children and adolescents, including subcortical volume, shape deformation (9), gyrification, and overall or regional gray matter volume (GMV) reduction (10, 11), but there are very few longitudinal research on cortical development. A longitudinal study observed persistent damage to human white matter development in HIV+ children, regardless of whether they received antiretroviral therapy or viral suppression at an early stage (12). Furthermore, many studies have used the emerging MRI technology to reveal the characteristics of brain structure, function and the influence of cognitive factors in children with HIV/AIDS, but the future impact of these developmental abnormalities are still unclear, brain regions vulnerable to HIV or antiretroviral drugs have also not been identified (13–15).

Some studies have shown that the developmental maturity of brain structure is consistent with the development of individual cognitive ability (16, 17). We hypothesized that HIV would have less influence on the development of brain regions related to primary functions such as sensory and motor domains, but have a greater impact on the development of brain regions related to advanced cognition such as decision-making and reasoning. We attempted to conduct cross-sectional and longitudinal studies through high-resolution structural MRI to observe which brain regions HIV may preferentially affect and to combine multiple domains of neuropsychological cognitive testing to observe the correlation between brain structural changes and cognitive ability.

## Materials and Methods

### Subjects

We recruited 16 HIV+ adolescents (mean age ± SD, 13.63 ± 1.83 years; range, 11–17 years; mean CD4 count ± SD, 558.87 ± 199.89 cells/mm^3^; range, 276–940), the viral load of them did not reach the lower limit of detection (<20 copies/ml). The presence of HIV+ children was confirmed by Western blot. We also recruited 25 age- and gender-matched HIV-Exposed Uninfected (HEU) subjects (mean age ± SD, 13.32 ± 1.62 years; range,11–17 years). All HIV+ adolescents were infected by mother-to-child transmission during pregnancy, childbirth or through breastfeeding. HEU subjects' fathers, mothers, or parents also suffered from HIV infections. The socioeconomic status, cultural background and ethnic background of the two communities were similar. Detailed population information and clinical measures are listed in Table 1. All subjects were enrolled from Training Centre of AIDS Prevention and Cure of Hubei Province. The inclusion criteria for HIV+ subjects included HIV acquisition during the fetal or neonatal period, currently treated with ART, and right-handed. For the control subjects, the inclusion criteria included confirmation of HIV negative status by ELISA and right-handedness.

**Table 1:** Demographics and results of neuropsychological tests.

	**HIV+ group**	**HEU group**	***p*-value**	**Cohen' *d*(ES)**	**Power**
Number of subjects (*n*)	16	25	–	–	–
Sex (male/female)	8/8	12/13	0.915	–	–
Age (years)	13.63 ± 1.83	13.32 ± 1.62	0.589	0.176	0.135
BMI (kg/m^2^)	18.27 ± 2.66	18.29 ± 3.06	0.873	0.008	0.053
Education (years)	7.31 ± 2.20	7.16 ± 1.51	0.799	0.081	0.081
Ethnicity (Han/Tujia)	15/1	24/1	0.904	–	–
Longitudinal data (*n*)	5 (31.25%)	7 (28.0%)	–	–	–
Mother-to-child transmission	16 (100%)	25 (100%)	–	–	–
CD4 count (cells/mm^3^)	558.87 ± 199.89	–	–	–	–
**COGNITIVE DOMAIN**					
Vocabulary/language (*n*)	4 (26.67%)	0 (0%)	0.17	–	–
Working memory/attention (*n*)	3 (23.08%)	0 (0%)	0.269	–	–
Executive/abstraction (*n*)	7 (46.67%)	0 (0%)	**0.015**	–	–
Memory/learning and recall (n)	1 (6.67%)	0 (0%)	0.733	–	–
Sensory perceptual/motor skills (*n*)	0 (0%)	0 (0%)	–	–	–

*HIV+, HIV-infected; HEU, HIV-exposed uninfected; ES, effect size; BMI, Body Mass Index, Values are n (% of total) or mean ± standard-deviation. Cognitive results of individuals in HIV+ group failed to follow the normal distribution test, thus we used the rank sum test. Significance at P < 0.05*.

Exclusion criteria for all subjects included those younger than 11 years of age or over 17 years of age, with acute medical illnesses, current or past medical or neurological diseases, psychiatric illnesses, mental retardation, current alcohol or drug abuse, HIV encephalopathy and opportunistic infections, MRI contraindications, claustrophobia, metabolic disorders or other brain diseases (not AIDS-related). We used the exclusion criteria, which included HIV-related encephalopathy to rule out space-occupying masses, other lesions or obvious cortical atrophy in the brain of HIV+ adolescents, so that we got the difference in anatomical gray matter covariance between the two groups. Only one individual in the HIV+ group was excluded because of age 7. For the control subjects, the exclusion criteria also included serious educational difficulties and a chronic medication other than asthma medication.

The study was approved by the Medical Ethics Committee of Zhongnan Hospital of Wuhan University, and a written and informed consent was made from all participants or their guardians in accordance with the Helsinki Declaration of 1975 (and as revised in 1983), following a complete description of the measurements. These methods were carried out in accordance with the approved guidelines and regulations.

### Neuropsychological Tests

In order to more comprehensively assess the cognitive abilities of the subjects, we selected five tests based on the Frascati criteria (21). (1) Word Semantics Test: to examine written language comprehension, especially at the level of sentences. (2) Verbal Working Memory Test (present audio): comes from the digital memory span subtest of Wechsler Intelligence Scale. (3) Wisconsin Card Sorting Test: to examine executive control capabilities. (4) Picture Memory Test. (5) Indicate the Midpoint Test of the Line Segment: focus on sensory perceptual and motor skills. We conducted all tests online using the professional “Multi-Dimensional Psychology” platform (http://www.dweipsy.com/lattice/). All these tests and MRI scans were carried out within a month of study enrollment for each participant.

### MRI Acquisition

High-resolution T1-weighted structural MRI scans were acquired on the 3.0 T scanner (Siemens, Prisma, Germany), which was stationed at the Department of Radiology, the Zhongnan Hospital of Wuhan University, using a multi-echo magnetization prepared rapid gradient echo (MPRAGE) pulse sequence (repetition time = 5,000 ms, echo time = 2.88 ms, inversion time = 700 ms, flip angle = 4°, slice thickness = 1.00 mm, and matrix size = 256 × 256) that yielded 176 axial slices with an in-plane resolution of 1.0 × 1.0 mm. We visually inspected the cerebral microbleeds foci measured by susceptibility-weighted imaging (SWI), and white matter hyperintensity by T2- fluid-attenuated inversion recovery (FLAIR) images through all the subjects. We also excluded any subject that exhibited obvious gray and white matter lesion imaged by SWI and FLAIR.

### Brain Morphometry Analysis

In this study, we analyzed two morphological brain measures, the gray matter volume [via voxel-based morphometric (VBM) analysis] and cortical thickness (CT) analysis. All data processing uses Statistical Parametric Mapping software (SPM12; Wellcome Department of Cognitive Neurology, London, UK; http://www.fl.ion.ucl.ac.uk/spm) and Computational Anatomy Toolbox (CAT12, http://dbm.neuro.uni-jena.de/vbm/) based on MATLAB (MathWorks, Natick, MA, USA). The data preprocessing mainly included: (1) experienced radiologists screen data with obvious abnormalities, excluding data with large artifacts and obvious lesions; (2) the original T1 images were manually reoriented to match the AC-PC plane, (3) segmented into gray matter, white matter, and cerebrospinal fluid using the standard unified segmented model (18, 19) in the CAT12, (4) nonlinearly normalized into standard Montreal Neurological Institute (MNI) space using a pediatric template for 12- to 18-year-old children from the Imaging Research Center at Cincinnati Children's Hospital Medical Center (CCHMC), (5) the normalized segmentations were then modulated to ensure that the relative volumes of gray matter were retained, (6) the modulated images were re-sampled to 1.5 × 1.5 × 1.5 mm^3^ and smoothed with an 8 mm full-width at half-maximum (FWHM) Gaussian kernel, (7) to control for deviations, we included an additional quality check based on heterogeneity measurements of the sample as implemented in CAT; using the covariance of voxel-based data to identify the outliers who were two or more standard deviations outside of the GMV sample distributions, and one patient and one control were excluded based on this criterion, (8) finally, exclude voxels with a gray matter value < 0.15 to eliminate the potential edge effects between the gray matter and white matter.

The CT was also computed by using the CAT12, all the parameters were set by default in the CAT12, except for the brain template using a pediatric template for 12- to 18-year-old children from the Imaging Research Center at Cincinnati Children's Hospital Medical Center (CCHMC) (https://irc.cchmc.org/software/pedbrain.php). Briefly, this automated method (20) allows for central surface reconstructions and CT measurement in one step, then the topological defects of cortical surface mesh were repaired by using a spherical harmonic method. Prior to the statistical analyses, the individual CT maps were smoothed by using a Gaussian filter with full-width at half-maximum of 15 mm.

For the longitudinal analysis of GMV and CT, we also used the CAT12 default parameters with the pediatric template and performed the analysis under the longitudinal analysis module to obtain subtle changes at the individual level between the two-time points (1 year before and after).

### Statistical Analysis

For clinical and behavioral data, statistical analysis was conducted using IBM SPSS version 20 (IBM SPSS Inc., Chicago, IL, USA) and G^*^Power 3.1.9.3. The significance threshold was set to *p* < 0.05. For the brain morphological parameters (gray matter volume and cortical thickness), cross-sectional between-group comparisons were tested in SPM12 using independent two-sample *t*-tests, with age and gender as covariates, longitudinal comparisons were carried by flexible design 2 × 2 analysis of variance (ANOVA). The significance threshold was set to *p* < 0.05 with a cluster level Family Wise Error (FWE) correction and threshold-free cluster enhancement (TFCE) multiple comparison-corrected.

## Results

### Demographics and Neuropsychological Tests

For demographic and clinical data, the HIV+ and HEU controls were comparable on age, gender, and education (*p* > 0.05, Table 1). For cognitive testing, the HIV+ group performed significantly worse in the Wisconsin card classification test (execution and abstraction functions) than their HEU controls (*p* = 0.015, Table 1). In addition, we also noted that 5 of the adolescents in the HIV+ group scored at least 2 items below the mean of the control group minus a standard deviation of one time, which can be diagnosed asymptomatic neurocognitive impairment according to Frascati criteria (21).

### Between-Group Comparison on Gray Matter Volume

**Figure 1:**
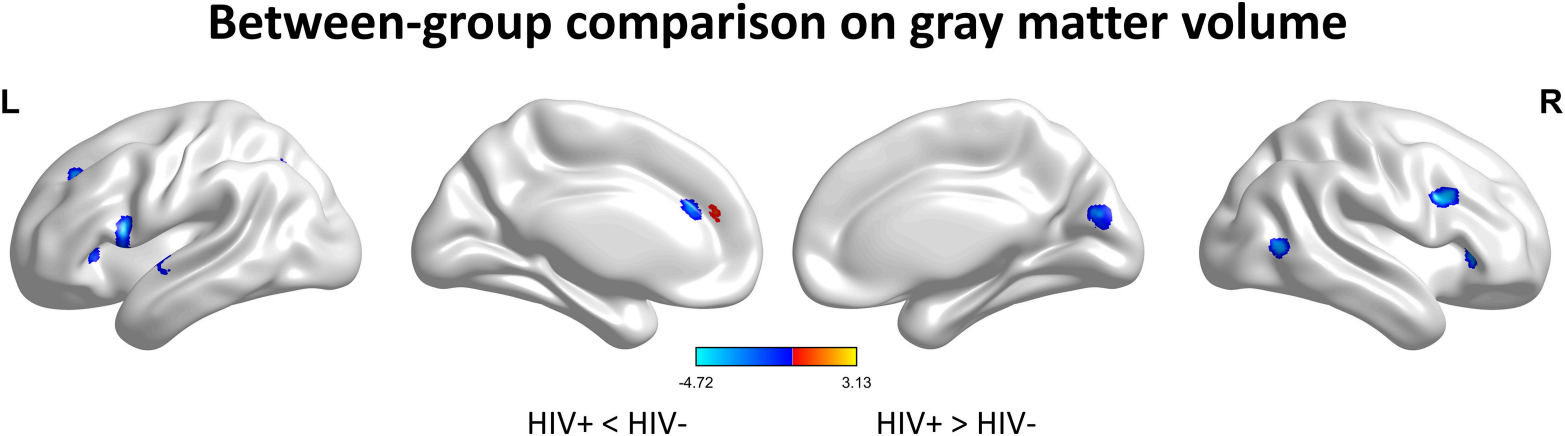
Between-group comparison on gray matter volume (GMV). Clusters showing significantly (corrected *P* < 0.05) lower (blue) and higher (red) GMV in HIV+ children compared to controls. But the red region isn't successfully positioned in the template. This figure was rendered using BrainNet Viewer (http://www.nitrc.org/projects/bnv/).

We first investigated the between-group differences on GMV by using VBM. This analysis revealed a significant reduction in the GMV across broad regions, including bilateral superior cerebellar, frontal, temporal, insular, angular regions, and right cuneus, in the HIV+ individuals, these results were corrected by FWE cluster level *p* < 0.05 (Table 2; Figure 1).

**Table 2:** Between-group comparison on gray matter volume.

**Contrast**	**Brain regions**	**Extent**	***t*-value**	**MNI Coordinates**		
				***x***	**y**	**z**
HIV + < HEU	Frontal_Inf_Tri_R	359	−4.7245	37.5	16.5	28.5
	Frontal_Inf_Oper_L	181	−3.8543	−48	12	13.5
	Temporal_Mid_R	158	−3.8214	45	−58.5	4.5
	Cerebelum_Crus1_L	334	−3.6533	−36	−69	−34.5
	Parietal_Inf_L	146	−3.5301	−27	−61.5	39
	Cerebelum_Crus1_R	378	−3.3279	39	−61.5	−37.5
	Cuneus_R	86	−3.2899	7.5	−81	18
	Insula_L	276	−3.251	−28.5	27	6
	Temporal_Pole_Sup_L	132	−3.1098	−51	6	−3
	Frontal_Mid_2_L	139	−3.0554	−31.5	19.5	60
	Frontal_Inf_Oper_L	124	−2.9471	−39	4.5	25.5
	Frontal_Sup_2_L	88	−2.9143	−18	33	39
	Insula_R	72	−2.9079	34.5	30	0
	Temporal_Sup_L	66	−2.7822	−52.5	−9	−3
	Angular_R	50	−2.7661	30	−60	40.5

*HIV+, HIV-infected; HEU, HIV-exposed uninfected; MNI, Montreal Neurological Institute; L, left; R, right. All regions were corrected by FWE cluster level p < 0.05*.

### Between-Group Comparison on Cortical Thickness

**Figure 2:**
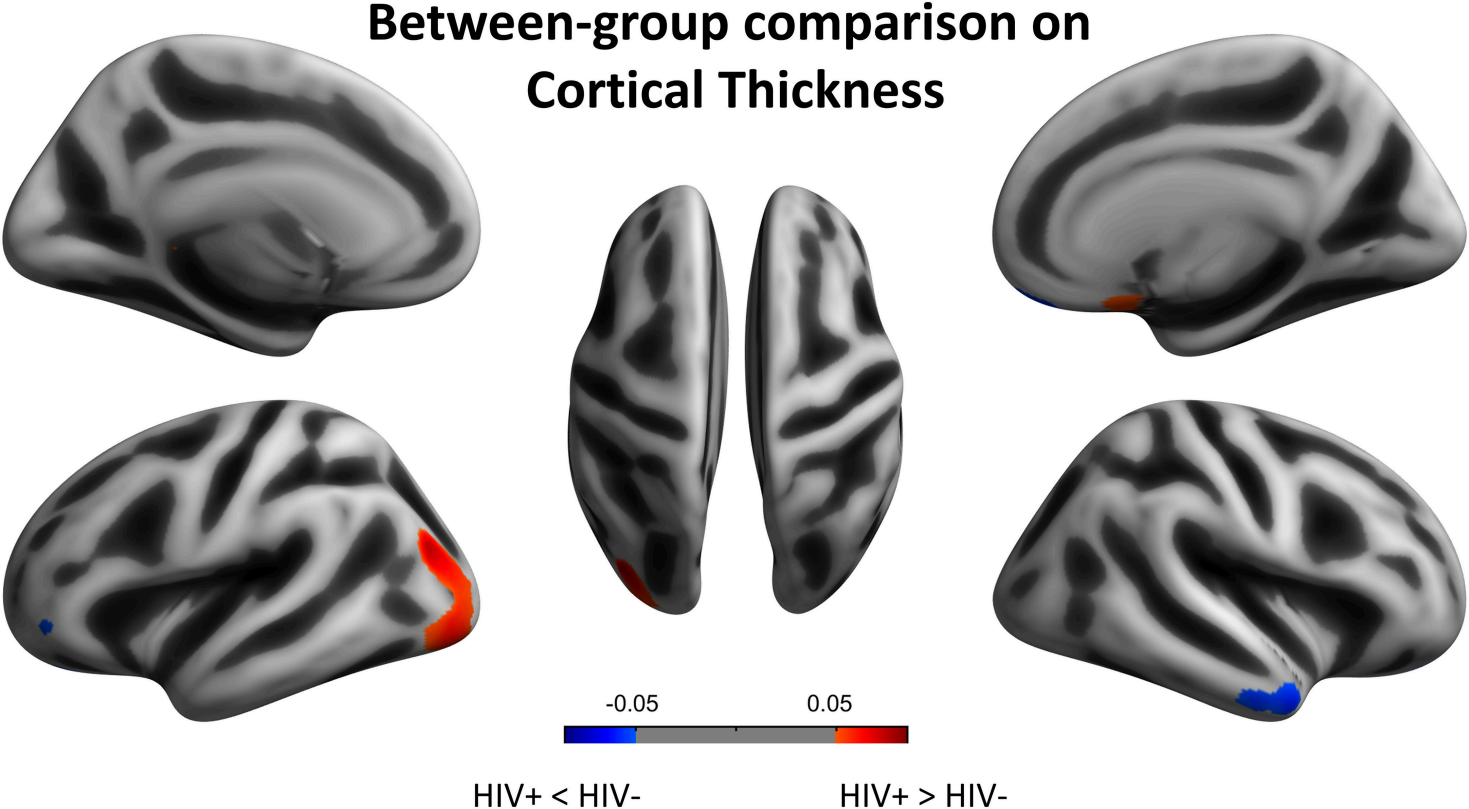
Between-group comparison on cortical thickness (CT). Clusters showing significantly (corrected *P* < 0.05) lower (blue) and higher (red) CT in HIV+ children compared to controls. These figures were rendered using BrainNet Viewer (http://www.nitrc.org/projects/bnv/).

We next reported the between-group differences on surface-based metrics of CT. Individuals with HIV showed significantly thicker cortices in left occipital (the middle and inferior gyri) and right olfactory sulcus, also, significantly thinner cortices in the temporal (the middle and the pole parts) and orbitofrontal regions. The results were corrected by FWE cluster level *p* < 0.05 (Table 3; Figure 2).

**Table 3:** Between-group comparison on cortical thickness.

**Contrast**	**Cortical regions**	**Side**	**Size**	***P*-value**
HIV +> HEU	42% G_occipital_middle 24% S_oc_middle_and_Lunatus 21% G_and_S_occipital_inf 9% Pole_occipital 2% G_pariet_inf-Angular 2%S_oc_sup_and_transversal	L	611	0.00960
	100% G_cingul-Post-ventral	L	1	0.04800
	42% S_orbital_med-olfact 38% G_rectus 15%G_subcallosal	R	79	0.04260
HIV+ < HEU	74% G_orbital 26%S_orbital_lateral	L	19	0.04740
	100% G_orbital	L	15	0.04640
	44% G_temporal_middle 32% Pole_temporal 12% S_temporal_inf 11% G_temp_sup-Lateral 1%S_temporal_sup	R	214	0.02880
	66% G_orbital 22% S_orbital_med-olfact 11% G_rectus 1%G_and_S_frontomargin	R	73	0.03380

*HIV+, HIV-infected; HEU, HIV-exposed uninfected; L, left; R, right. All regions were corrected by FWE cluster level p < 0.05*.

### Longitudinal Changes in Gray Matter Volume

Based on the cross-sectional results, an important aim of this study is to investigate the longitudinal brain develop trajectory. Using a 2 × 2 ANOVA, we then contrasted the development patterns between the two groups.

**Figure 3:**
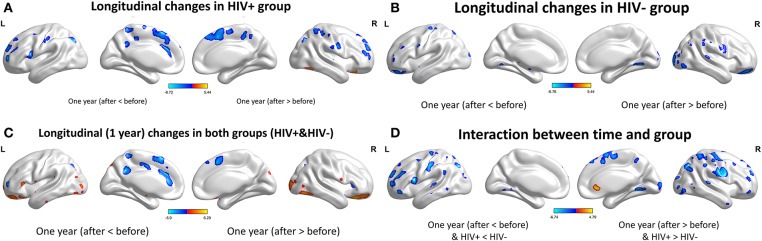
Longitudinal changes on gray matter volume (GMV) after 1 year. Clusters showing significantly (corrected *P* < 0.05) lower (blue) and higher (red) GMV changes after 1 year. **(A)** Clusters showing many lower and few higher GMV regions in HIV+ group children. **(B)** Clusters only showing significantly lower GMV regions in HEU group children. **(C)** Put together the two groups for statistical analysis and showing significantly changes of GMV after 1 year. **(D)** In assessing the time and interactions between the two groups, clusters showing widely areas of GMV significantly reduced. These figures were rendered using BrainNet Viewer (http://www.nitrc.org/projects/bnv/).

For the GMV, generally, the HIV+ group showed changes in GMV over a wider range of brain regions and was dominated by a significant decrease in GMV after 1 year. These regions mainly included decreased GMV in the bilateral frontal, supplementary, parietal, and occipital regions; and increased GMV in the right inferior temporal, inferior occipital and orbital frontal cortex (OFC) regions (Table 4; Figure 3A).

**Table 4:** Volume change of gray matter in HIV+ group after 1 year.

**Contrast**	**Brain regions**	**Extent**	***t*-value**	**MNI coordinates**		
				**x**	**y**	**z**
Positive	Temporal_Inf_R	144	5.440	44	−56	−14
	Occipital_Inf_R	144	4.362	51	−75	−14
	OFCmed_R	134	5.293	21	27	−20
Negative	Frontal_Mid_2_L	306	−8.717	−30	54	6
	Occipital_Sup_R	292	−7.799	27	−69	42
	Supp_Motor_Area_R	2409	−7.215	5	15	50
	Frontal_Sup_Medial_R	2409	−6.253	6	38	51
	Parietal_Sup_R	172	−6.944	38	−50	56
	Precuneus_L	755	−6.851	−8	−47	63
	Paracentral_Lobule_R	755	−4.995	12	−35	48
	Parietal_Inf_L	315	−6.177	−44	−42	51
	Postcentral_L	315	−4.796	−53	−15	36
	Frontal_Sup_2_L	292	−5.973	−18	54	26
	Parietal_Inf_L	281	−5.877	−27	−69	42
	Frontal_Inf_Oper_L	296	−5.857	−50	8	15
	Postcentral_R	113	−5.798	56	−6	29
	Frontal_Sup_2_R	442	−5.697	24	54	27
	Frontal_Mid_2_R	442	−4.432	39	50	14
	Postcentral_R	286	−5.620	47	−35	54
	Cingulate_Post_L	96	−5.516	−6	−50	32
	Precuneus_R	95	−5.365	8	−50	51
	Frontal_Mid_2_L	154	−5.351	−30	47	21
	SupraMarginal_R	69	−5.146	65	−17	27
	Cerebelum_Crus1_L	50	−5.038	−45	−48	−41
	Supp_Motor_Area_R	335	−4.992	3	−11	62
	Frontal_Sup_2_R	81	−4.660	15	47	41
	Temporal_Sup_R	51	−4.435	56	−14	8
	Cerebelum_4_5_L	57	−4.291	−6	−47	−5
	Parietal_Inf_L	54	−4.237	−53	−44	36
	Frontal_Sup_2_L	50	−4.075	−18	42	44
	Precentral_L	71	−4.003	−38	0	60

*HIV+, HIV-infected; MNI, Montreal Neurological Institute; L, left; R, right. All regions were corrected by FWE cluster level p < 0.05*.

The HEU group showed only areas with significantly reduced GMV after 1 year, mainly in the bilateral temporal lobe, left inferior frontal gyrus, right anterior OFC, and right parietal lobe (postcentral, angular, and superior parietal gyrus) and right occipital lobe, part of the basal ganglia nuclei (putamen, thalamus), bilateral cerebellum (Table 5; Figure 3B).

**Table 5:** Volume change of gray matter in HEU group after 1 year.

**Contrast**	**Brain regions**	**Extent**	***t*-value**	**MNI coordinates**		
				**x**	**y**	**z**
Negative	Temporal_Pole_Sup_L	5516	−6.760	−60	8	−5
	Frontal_Inf_Oper_L	5516	−5.867	−54	20	32
	Postcentral_R	4111	−5.767	65	0	30
	Temporal_Pole_Mid_R	4111	−5.650	50	24	−27
	Temporal_Pole_Sup_R	4111	−5.417	62	11	−6
	OFCant_R	2130	−5.588	47	53	−14
	Precuneus_R	98	−5.308	2	−66	62
	Supp_Motor_Area_R	170	−5.105	9	2	75
	Angular_R	1266	−5.008	56	−68	35
	Parietal_Sup_R	1266	−4.506	35	−74	53
	Parietal_Sup_R	1266	−4.469	51	−39	59
	Frontal_Mid_2_R	357	−4.989	45	12	56
	Precentral_R	357	−3.925	42	−8	66
	Thalamus_R	413	−4.890	15	−20	5
	Lingual_L	1601	−4.130	−15	−66	−9
	Cerebelum_4_5_L	1601	−3.965	−11	−50	−23
	Occipital_Sup_R	1146	−4.669	23	−102	9
	Occipital_Mid_R	1146	−4.498	33	−90	26
	Occipital_Mid_R	1146	−4.086	39	−93	2
	Calcarine_L	123	−4.591	5	−98	0
	Temporal_Pole_Mid_L	269	−4.044	−20	15	−39
	Temporal_Sup_R	79	−4.429	66	−53	20
	Occipital_Inf_R	70	−4.388	44	−78	−9
	Thalamus_L	386	−4.288	−17	−27	2
	Putamen_R	940	−4.349	27	12	−8
	Putamen_R	940	−4.255	30	−5	11
	Precentral_L	306	−4.309	−30	−21	69
	Lingual_R	186	−4.254	15	−65	−6
	Temporal_Inf_L	72	−4.231	−69	−33	−20
	Cerebelum_4_5_R	553	−4.139	18	−45	−26
	Parietal_Inf_L	70	−4.125	−38	−63	51
	Occipital_Sup_L	77	−3.911	−18	−98	15

*HEU, HIV-exposed uninfected; MNI, Montreal Neurological Institute; L, left; R, right. All regions were corrected by FWE cluster level p < 0.05*.

The two groups were put together for statistical analysis and showed that after 1 year, the GMV of multiple brain regions increased significantly, mainly including bilateral frontal lobes (the right OFC, the left inferior frontal cortex, the right precentral cortex, etc), and the right temporal lobe, bilateral upper cerebellar and parietal-occipital lobes, right amygdala, and thalamus; areas of markedly reduced GMV mainly include right medial OFC, left supplemental motor area, left cingulate gyrus, and temporal-occipital cortex (Table 6; Figure 3C). In assessing the time and interactions between the two groups, it was shown that the volume of gray matter in a wide area was significantly reduced, mainly including the bilateral parietal lobes (above gyrus, supramarginal gyrus, and precuneus), and bilateral multiple other lobes; significant gray matter volume increase only in the right cingulate anterior (Table 7; Figure 3D).

**Table 6:** Volume change of gray matter in HIV+ and HEU groups after 1 year.

**Contrast**	**Brain regions**	**Extent**	***t*-value**	**MNI coordinates**		
				**x**	**y**	**z**
Positive	OFCpost_R	3035	5.829	23	23	−27
	Temporal_Inf_R	1626	4.445	56	−45	−29
	Cerebelum_6_R	1626	3.393	18	−62	−30
	Temporal_Pole_Sup_R	1996	5.278	57	18	−14
	Precentral_R	1996	4.207	66	5	18
	Temporal_Sup_R	1996	4.155	66	0	−5
	Occipital_Inf_R	630	5.100	50	−75	−12
	Temporal_Inf_R	630	4.142	45	−56	−14
	Occipital_Mid_L	2366	4.276	−33	−87	2
	Parietal_Sup_L	2366	4.011	−29	−72	57
	Frontal_Inf_Oper_L	1136	4.559	−54	20	33
	Frontal_Inf_Tri_L	1136	4.434	−59	26	6
	Frontal_Mid_2_L	1136	4.238	−48	50	6
	Amygdala_R	931	3.584	32	−5	−14
	Cerebelum_4_5_L	1443	4.258	−29	−27	−33
	Frontal_Sup_Medial_L	556	3.275	0	68	12
	Temporal_Sup_R	212	3.985	66	−51	23
	OFCant_L	197	3.831	−21	30	−18
	Occipital_Sup_R	463	3.819	26	−101	9
	Occipital_Inf_R	463	3.105	30	−87	−8
	Cerebelum_9_R	129	3.530	18	−41	−53
	Putamen_R	249	3.528	26	12	−8
	Parietal_Sup_L	58	3.528	−24	−57	74
	Postcentral_L	69	3.522	−36	−32	72
	Occipital_Mid_R	141	3.506	36	−89	24
	Frontal_Sup_Medial_L	114	3.476	−17	66	−2
	Parietal_Inf_R	54	3.438	42	−63	57
	Thalamus_L	74	3.286	−20	−27	0
Negative	Frontal_Mid_2_L	247	−5.901	−29	54	6
	Precuneus_L	100	−5.162	−8	−47	63
	Supp_Motor_Area_L	1270	−4.985	−6	20	56
	Cingulate_Mid_L	1270	−4.004	0	−2	39
	Cingulate_Mid_L	1270	−3.695	−11	−20	47
	Temporal_Sup_L	115	−4.479	−50	−42	18
	Cingulate_Post_L	248	−4.388	−6	−48	32
	Occipital_Mid_L	291	−4.161	−26	−62	38
	Cerebelum_8_R	57	−4.073	12	−69	−45
	Insula_R	63	−3.784	38	26	3
	Occipital_Sup_R	174	−3.739	26	−69	42
	Temporal_Sup_L	141	−3.438	−42	−15	−6
	Cerebelum_9_R	54	−3.395	2	−53	−56
	Temporal_Sup_R	51	−3.256	44	−8	−8
	OFCmed_R	3035	3.958	18	26	−24

*HIV+, HIV-infected; HEU, HIV-exposed uninfected; MNI, Montreal Neurological Institute. All regions were corrected by FWE cluster level p < 0.05*.

**Table 7:** Interactions between groups and time points of gray matter volume.

**Contrast**	**Brain regions**	**Extent**	***t*-value**	**MNI coordinates**		
				**x**	**y**	**z**
Positive	Cingulate_Ant_R	100	4.936	5	27	−3
Negative	Occipital_Sup_R	676	−6.745	27	−69	44
	Frontal_Sup_2_L	2897	−6.375	−27	56	8
	Frontal_Mid_2_L	2897	−5.558	−44	41	29
	Frontal_Inf_Tri_L	2897	−4.869	−56	35	12
	SupraMarginal_R	4756	−6.233	65	−17	27
	Parietal_Sup_R	4756	−5.901	38	−50	56
	Postcentral_R	4756	−5.317	54	−26	56
	Vermis_10	221	−5.884	−2	−45	−38
	Parietal_Inf_L	84	−5.858	−44	−42	51
	Temporal_Pole_Sup_L	774	−5.560	−57	9	−6
	Frontal_Inf_Oper_L	774	−4.651	−51	9	15
	Occipital_Inf_R	213	−5.559	33	−83	−11
	Supp_Motor_Area_R	549	−5.552	9	2	75
	Parietal_Inf_L	428	−5.363	−51	−26	47
	Frontal_Sup_Medial_R	494	−5.327	9	38	56
	Cerebelum_4_5_L	902	−5.290	−6	−47	−5
	Lingual_L	902	−4.408	−15	−63	−12
	Cerebelum_Crus1_L	168	−5.261	−29	−80	−26
	OFCant_R	239	−5.039	45	51	−15
	Precuneus_R	420	−4.979	8	−50	51
	Precentral_L	436	−4.966	−41	0	62
	Frontal_Sup_Medial_L	131	−4.938	−6	54	44
	Supp_Motor_Area_R	124	−4.898	6	15	50
	Frontal_Sup_2_R	287	−4.855	27	59	24
	Angular_R	280	−4.850	53	−66	35
	Cerebelum_4_5_R	481	−4.829	9	−60	−5
	Frontal_Mid_2_R	241	−4.765	39	54	18
	Parietal_Inf_L	380	−4.732	−38	−62	53
	Precuneus_R	63	−4.695	3	−66	60
	Cerebelum_Crus2_R	443	−4.590	42	−78	−44
	Temporal_Mid_R	113	−4.542	65	−2	−21
	Temporal_Sup_L	171	−4.511	−65	−27	6
	SupraMarginal_L	355	−4.497	−59	−50	26
	Temporal_Sup_R	85	−4.447	65	−8	8
	Occipital_Sup_R	126	−4.441	24	−93	20
	Frontal_Sup_2_R	77	−4.410	23	12	68
	Temporal_Mid_R	402	−4.355	65	−42	9
	Temporal_Mid_R	335	−4.330	53	−66	8
	Parietal_Sup_L	66	−4.289	−21	−66	59
	Occipital_Mid_L	69	−4.262	−29	−87	21
	Precentral_L	182	−4.256	−27	−23	71
	Frontal_Sup_2_L	100	−4.254	−20	44	45
	Occipital_Mid_R	87	−4.246	38	−95	0
	Temporal_Pole_Mid_R	193	−4.211	51	9	−35
	Frontal_Sup_Medial_R	83	−4.103	11	69	2
	Temporal_Mid_L	80	−4.092	−60	−54	−5
	Thalamus_R	137	−3.943	17	−18	8
	Thalamus_L	214	−3.885	−6	−6	8
	Frontal_Inf_Tri_R	122	−3.882	53	38	17

*Two groups = HIV-infected group and HIV-exposed uninfected group, MNI, Montreal Neurological Institute; L, left; R, right. All regions were corrected by FWE cluster level p < 0.05*.

### Longitudinal Changes on Cortical Thickness

**Figure 4:**
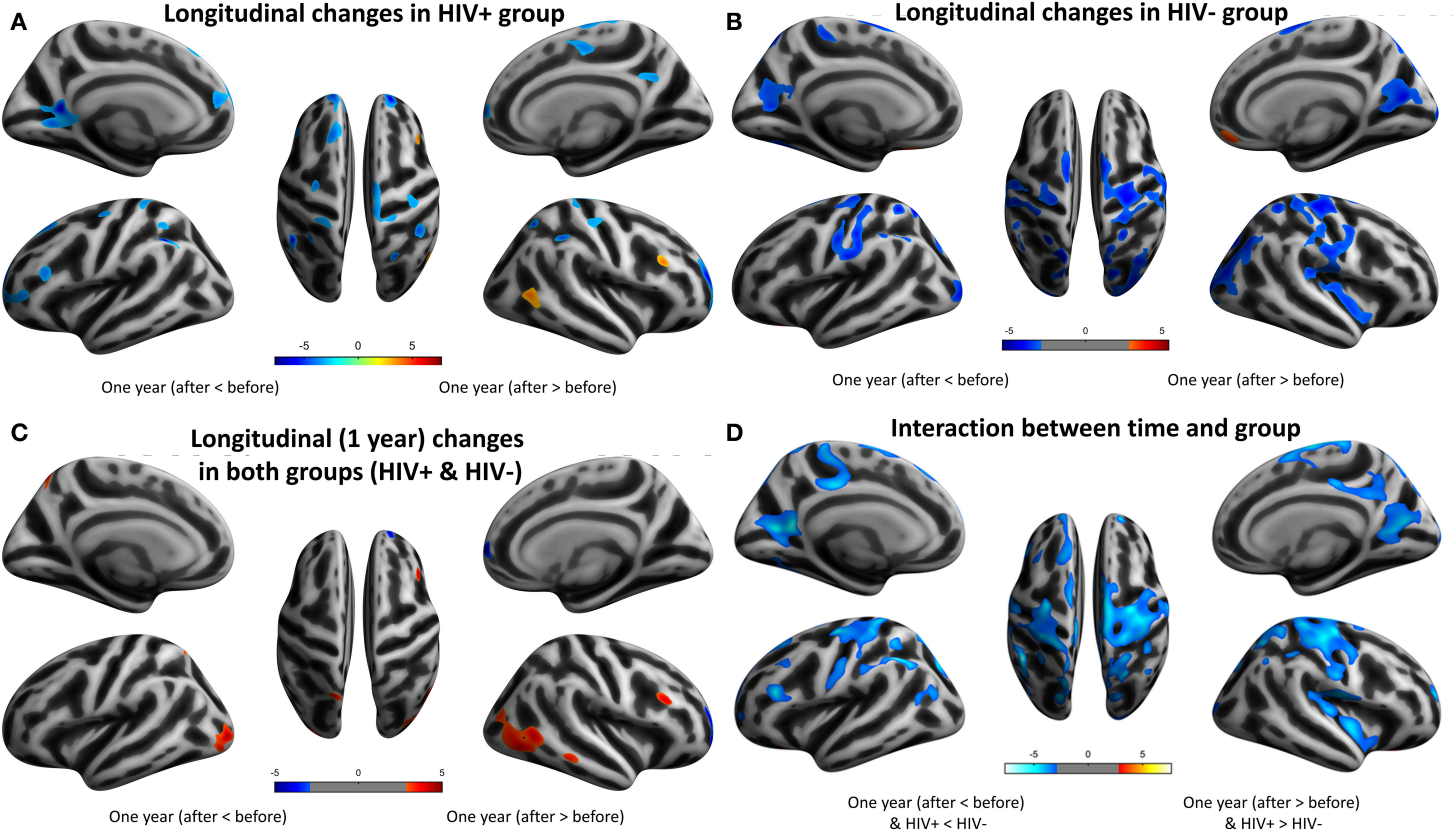
Longitudinal changes on cortical thickness (CT) after 1 year. Clusters showing significantly (corrected *P* < 0.05) lower (blue) and higher (red) CT changes after 1 year. **(A)** Clusters showing lower and higher CT regions in HIV+ group children. **(B)** Clusters showing significantly lower and higher CT regions in HEU group children. **(C)** Put together the two groups for statistical analysis and showing significantly lower regions more obviously than higher regions of CT after 1 year. **(D)** In assessing the time and interactions between the two groups, clusters showing widely areas of CT significantly reduced.

For the cortical measures, the longitudinal results showed extensive cortical thinning in both the HIV+ and HEU groups after 1 year. In the HIV+ group, the bilateral cingulate gyrus, the postcentral gyrus, the parietal lobe and the left superior frontal gyrus, the calcarine, the angular gyrus, the precentral gyrus, the inferior frontal gyrus, the right paracentral lobes, the frontal pole, the central sulcus, and the inferior parietal lobule were displayed (Table 8; Figure 4A).

**Table 8:** Cortical thickness change in HIV+ group after 1 year.

**Contrast**	**Cortical regions**	**Side**	**Size**	***P*-value**
Positive	92% G_oc-temp_med-Parahip 8%S_collat_transv_ant	L	490	0.00287
	28% S_occipital_ant 22% S_temporal_inf 17% G_and_S_occipital_inf 13% S_temporal_sup 12% G_temporal_middle 4% G_occipital_middle 4%G_temporal_inf	R	1205	0.00032
	75% S_front_inf 25%G_front_middle	R	306	0.00029
Negative	56% G_front_sup 15% G_front_middle 8% G_and_S_frontomargin 6% G_and_S_cingul-Ant 5% G_and_S_transv_frontopol 4% S_orbital_lateral 3% S_front_sup 2% S_front_inf 1%S_front_middle	L	3546	0.00006
	66% S_calcarine 11% G_cingul-Post-ventral 8% S_parieto_occipital 8% G_oc-temp_med-Lingual 6%G_precuneus	L	2371	0.00000
	82% G_postcentral 8% S_postcentral 8% S_central 2%G_and_S_paracentral	L	851	0.00139
	48% G_pariet_inf-Angular 27% S_intrapariet_and_P_trans 19% G_pariet_inf-Supramar 7%S_postcentra	L	847	0.00000
	55% G_precentral 45%S_precentral-sup-part	L	671	0.00043
	77% S_front_inf 17% G_front_middle 5% G_front_inf-Opercular 2%G_front_inf-Triangul	L	482	0.00036
	51%G_cingul-Post-dorsal 36% S_cingul-Marginalis 12% G_and_S_cingul-Mid-Post 2% G_precuneus	L	335	0.00103
	56% S_intrapariet_and_P_trans 26% G_parietal_sup 18%S_postcentral	L	325	0.00095
	28% G_and_S_paracentral 26% G_front_sup 18% G_postcentral 17% S_central 10%G_precentral	R	2247	0.00085
	27% G_and_S_transv_frontopol 23% G_front_middle 21% G_front_sup 13% G_and_S_frontomargin 13% S_front_middle 2%G_and_S_cingul-Ant	R	1644	0.00000
	54% S_central 26% G_precentral 18%G_postcentral	R	1271	0.00173
	69% G_and_S_cingul-Mid-Post 31%G_front_sup	R	750	0.00070
	55% S_postcentral 40% S_interm_prim-Jensen 4%S_intrapariet_and_P_trans	R	688	0.00011
	83% S_subparietal 13% G_precuneus 4%G_cingul-Post-dorsal	R	581	0.00130
	100%S_intrapariet_and_P_trans	R	408	0.00006

*HIV+, HIV-infected; L, left; R, right. All regions were corrected by FWE cluster level p <0.05*.

The HEU group showed significant cortical thinning mainly around the bilateral central sulcus and mainly in the frontal lobe, left occipital lobe and the right temporal lobe, significantly thickened cortex mainly in the left olfactory sulcus, orbital gyrus and the right inferior orbital sulcus and the right gyrus rectus (Table 9; Figure 4B).

**Table 9:** Cortical thickness change in HEU group after 1 year.

**Contrast**	**Cortical regions**	**Side**	**Size**	***P*-value**
Positive	51% S_orbital_med-olfact 34% G_orbital 11% S_orbital-H_Shaped 5%G_rectus	L	619	0.00013
	54% S_suborbital 32% G_rectus 13% G_and_S_cingul-Ant 2%G_front_sup	R	449	0.00073
Negative	41% G_postcentral 32% S_central 16% S_postcentral 7% G_pariet_inf-Supramar 3%G_and_S_subcentral	L	5583	0.00055
	68% G_parietal_sup 10% G_occipital_sup 8% G_precuneus 6% S_intrapariet_and_P_trans 5% S_oc_sup_and_transversal 3%G_and_S_paracentral	L	2688	0.00004
	53% S_parieto_occipital 24% S_calcarine 15% G_precuneus 6% G_cuneus 1%G_cingul-Post-dorsal	L	2637	0.00085
	43% G_occipital_middle 29% Pole_occipital 20% S_oc_middle_and_Lunatus 7% S_oc_sup_and_transversal 1%G_and_S_occipital_inf	L	2249	0.00049
	50% G_precentral 43% S_central 6%S_precentral-sup-part	L	1875	0.00086
	55% G_pariet_inf-Angular 31% G_pariet_inf-Supramar 10% S_postcentral 2% G_occipital_middle 2%S_oc_sup_and_transversal	L	1728	0.00169
	58%S_cingul-Marginalis 18%G_and_S_paracentral 15%G_and_S_cingul-Mid-Post 4% G_cingul-Post-dorsal 2% G_front_sup 2%G_precuneus	L	1657	0.00109
	100%G_front_sup	L	1163	0.00064
	53%G_oc-temp_lat-fusifor 32%S_oc-temp_med_and_Lingual 9% G_oc-temp_med-Lingual 6%S_collat_transv_post	L	853	0.00052
	100% G_parietal_sup	L R	577 29750	0.00044 0.00024
	10%G_postcentral 9% S_central 9% G_precentral 6% G_parietal_sup 6% S_postcentral 5% G_pariet_inf-Supramar 5% G_occipital_middle 5% S_circular_insula_inf 5% Lat_Fis-post 5% Pole_occipital			
	5% G_and_S_subcentral 5% G_front_sup 4% G_occipital_sup 4% G_pariet_inf-Angular 3% S_oc_sup_and_transversal 2% G_Ins_lg_and_S_cent_ins 2% G_temp_sup-Plan_tempo 2% S_oc_middle_and_Lunatus 2% G_precuneus 1% G_insular_short 1%G_temp_sup-G_T_transv			
	48% S_parieto_occipital 27% S_calcarine 21% G_cuneus 3%G_precuneus	R	2817	0.00030
	61% G_temp_sup-Lateral 24% G_temp_sup-G_T_transv 10% S_temporal_transverse 4%G_temp_sup-Plan_polar	R	522	0.00204
	100% G_and_S_paracentral	R	305	0.00246
	62% G_front_middle 38%S_front_inf	R	302	0.00156

*HEU, HIV-exposed uninfected; L, left; R, right. All regions were corrected by FWE cluster level p < 0.05*.

The two groups were put together for statistical analysis, the areas of the cortical thickening were more obvious, mainly in the bilateral middle occipital gyrus, the parietal lobe (angle gyrus), the frontal lobe (near the right inferior frontal gyrus) and the right middle temporal gyrus. Cortical thinning was mainly in the right frontal lobe (Table 10; Figure 4C). In the evaluation of the time and the interaction between the two groups, the cortical thinning in a wide area was shown, mainly around the bilateral central sulcus, the insular, the frontal lobe (mainly in the middle gyrus), the cingulate sulcus. Significant cortical thickening only showed in bilateral orbital gyrus and the right parahippocampal gyrus (Table 11; Figure 4D).

**Table 10:** Cortical thickness change in HIV+ and HEU groups after 1 year.

**Contrast**	**Cortical regions**	**Side**	**Size**	***P*-value**
Positive	39% S_oc_middle_and_Lunatus 31% G_occipital_middle 15% Pole_occipital 14%G_and_S_occipital_inf	L	1687	0.00011
	66% G_parietal_sup 34%G_precuneus	L	820	0.00070
	32% G_occipital_middle 17% S_occipital_ant 12% S_oc_middle_and_Lunatus 12% G_and_S_occipital_inf 10% S_temporal_inf 9% G_temporal_middle 3% G_temporal_inf 3% S_temporal_sup 2%S_oc_sup_and_transversal	R	2912	0.00021
	99% G_pariet_inf-Angular	R	632	0.00253
	70% S_front_inf 30%G_front_middle	R	418	0.00023
	49% G_front_inf-Opercular 25% Lat_Fis-ant-Vertical 24% S_circular_insula_sup 1%G_front_inf-Triangul	R	361	0.00114
	100% G_temporal_middle	R	359	0.00096
Negative	44% G_and_S_transv_frontopol 26% G_and_S_frontomargin 23% G_front_middle 7%S_front_middle	R	764	0.00037
	59% G_front_sup 30% G_and_S_cingul-Ant 11%G_and_S_transv_frontopol	R	435	0.00011

*HIV+, HIV-infected, HEU, HIV-exposed uninfected; L, left; R, right. All regions were corrected by FWE cluster level p < 0.05*.

**Table 11:** Interactions between groups and time points of cortical thickness.

**Contrast**	**Cortical regions**	**Side**	**Size**	***P*-value**
Positive	57% G_orbital 23% S_orbital_med-olfact 20%S_orbital-H_Shaped	L	261	0.00025
	52% G_orbital 34% S_orbital-H_Shaped 15%S_orbital_med-olfact	R	810	0.00156
	92% G_oc-temp_med-Parahip 8%S_oc-temp_med_and_Lingual	R	207	0.00184
Negative	13% G_postcentral 12% S_central 11% G_parietal_sup 9% S_calcarine 8% G_pariet_inf-Angular 8% S_parieto_occipital 8% G_pariet_inf-Supramar 7% G_precentral 4% S_postcentral 4% S_temporal_sup 3% G_precuneus 3% S_intrapariet_and_P_trans 2% S_precentral-sup-part 2% G_occipital_sup 2% G_and_S_paracentral 1%G_cuneus	L	18597	0.00000
	90% G_front_sup 6% G_front_middle 3%S_front_sup	L	3533	0.00038
	40% S_cingul-Marginalis 18% G_and_S_paracentral 12% G_and_S_cingul-Mid-Post 11% G_precuneus 9% S_subparietal 7% G_cingul-Post-dorsal 4%G_front_sup	L	3215	0.00014
	54% S_central 18% G_precentral 9% G_postcentral 6% S_precentral-inf-part 6% G_and_S_subcentral 4% S_precentral-sup-part 2%G_front_middle	L	2387	0.00066
	31% S_circular_insula_inf 25% S_circular_insula_sup 20% S_circular_insula_ant 15% G_insular_short 10%G_Ins_lg_and_S_cent_ins	L	1696	0.00139
	48% S_front_inf 25% G_front_inf-Opercular 19% G_front_middle 7%G_front_inf-Triangul	L	1067	0.00008
	42% G_front_middle 33% G_and_S_frontomargin 15% S_orbital_lateral 5% S_front_inf 3% S_front_middle 3%G_orbital	L	721	0.00088
	54% S_intrapariet_and_P_trans 33% G_parietal_sup 13%S_postcentral	L	669	0.00045
	44% S_oc-temp_med_and_Lingual 37% G_oc-temp_lat-fusifor 19%G_oc-temp_med-Lingual	L	585	0.00234
	99% G_front_middle 1%S_front_sup	L	467	0.00177
	23% S_central 18% G_postcentral 16% G_precentral 13% G_front_sup 12% S_postcentral 8% G_and_S_paracentral 4% G_pariet_inf-Supramar 3% S_precentral-sup-part 1%G_and_S_cingul-Mid-Post	R	17427	0.00005
	26% S_circular_insula_inf 22% Lat_Fis-post 17% G_and_S_subcentral 8% G_Ins_lg_and_S_cent_ins 7% G_temp_sup-G_T_transv 6% G_insular_short 4% G_front_inf-Opercular 2% S_temporal_transverse 2% G_temp_sup-Lateral 2% S_circular_insula_ant 1%G_temp_sup-Plan_tempo	R	7725	0.00017
	27% S_subparietal 21% S_parieto_occipital 21% S_calcarine 9% G_precuneus 9% G_cuneus 5% G_cingul-Post-dorsal 4% S_cingul-Marginalis 2% G_oc-temp_med-Lingual 2%G_and_S_cingul-Mid-Post	R	5913	0.00019
	66% G_parietal_sup 20% S_intrapariet_and_P_trans 14% G_precuneus 1%S_postcentral	R	3429	0.00013
	49% Pole_occipital 17% G_occipital_middle 14% S_oc_sup_and_transversal 8% G_pariet_inf-Angular 6% G_occipital_sup 4% S_oc_middle_and_Lunatus 2%S_temporal_sup	R	2109	0.00176
	79% G_occipital_sup 8% S_oc_sup_and_transversal 5% G_cuneus 4% S_intrapariet_and_P_trans 3% G_parietal_sup 1%S_parieto_occipital	R	949	0.00037
	64% G_front_middle 28% S_front_middle 7% G_and_S_transv_frontopol 1%G_front_sup	R	408	0.00012
	61% G_oc-temp_lat-fusifor 39%S_oc-temp_med_and_Lingual	R	368	0.00124
	100%G_pariet_inf-Angular	R	362	0.00075
	80% G_front_middle 20%S_front_inf	R	226	0.00209

*Two groups = HIV-infected group and HIV-exposed uninfected group; L, left; R, right. All regions were corrected by FWE cluster level p < 0.05*.

### The Relationship Between Brain Morphological Metrics and Behavior

Finally, we analyzed the association between behavioral performance and brain morphological measures in HIV+ adolescents. Because there was a significant difference in Wisconsin Card Classification Test between the two groups, we performed a nonparametric correlation analysis on the Wisconsin Card Classification scores of the HIV+ group and the morphological measures with significant between-group differences on the cross-sectional analysis. For the GMV, the gray matter volume of the bilateral cerebellum in the HIV+ group was significantly positively correlated with the Wisconsin Card Classification Test score (*r* = 0.681, *p* = 0.005). For the CT, the cortical thickness of HIV+'s right rostral middle frontal (*r* = −0.646, *p* = 0.009) and right superior frontal gyri was significantly negatively correlated with the Wisconsin Card Classification Test score.

## Discussion

Cross-sectionally, we found a wide reduction in cortical volume and changes in cortical thickness with thinning of the right temporal lobe and thickening of the left occipital lobe in HIV+ individuals during adolescence. Longitudinally, the reduction in gray matter volume and thinning of the cortex in HIV+ individuals occurred mainly in different functional areas of the frontal and parietal lobe, which was significantly different from the pattern of their HEU controls.

Cross-sectionally, the alterations in the advanced cortices including the OFC and primary sensorimotor cortices as found here are generally consistent with our hypothesis and earlier reports (22, 23). The HIV virus can rapidly enter the central nervous system (CNS) and cause encephalitis within a few days after the exposure (24). In the early stages of the disease, HIV induces the CNS inflammatory T-cell response with vasculitis and leptomeningitis, directly damage oligodendrocytes, neurons, and white matter (25). The same process can be observed in the cerebral cortex of AIDS patients, thereby inducing neuronal apoptosis. Immunoreactive gliosis and neuronal loss can even cause diffuse poliodystrophy (26, 27). These pathological changes should explain why our GMV cross-sectional results only show a decrease in gray matter volume in multiple brain regions, mainly in the bilateral frontal, temporal, insular, and cerebellar cortical regions.

A major novelty of this study is the using of longitudinal structural brain data to examine brain development during early puberty after HIV exposure. Longitudinally, we found that the brain maturation in HIV-infected and non-HIV-infected children follows a general pattern, but unique changes have taken place in terms of developmental speed, spatial range, etc. The human brain decreases its gray matter in late childhood or adolescence in typical healthy development (28, 29). Our longitudinal VBM results also showed this trend, however, the GMV loss pattern of HIV+ children are significantly different from that of the controls over 1 year, the topographies of this loss including the right frontal, superior parietal, and sensorimotor regions. Changes in the OFC have been suggested with pronounced negative emotions (30). The difference in volume changes in the right OFC after 1 year suggests that this brain area may be one of the key brain areas affected by HIV. The OFC is an important limbic brain region responsible for emotion, decision making and reward evaluation, which collectively with basal ganglia, amygdala and the medial prefrontal cortex form an limbic-emotion circuit (31). It is generally believed that the functional imbalance of this circuit is related to the pathogenesis of obsessive-compulsive disorder (32, 33).

The neuroanatomical features are significantly controlled over genetic and environmental factors. GMV is a function of surface area and cortical thickness, the cortical surface area has been reported to more likely to be regulated by a genetic factor than the cortical thickness (34). A human study has shown that a decrease in synaptic density in healthy children between the ages of 2 and 16 years is accompanied by a slight decrease in neuronal density (35). We can consider that thinning of the cortex during adolescence represents the concurrent process of synapses, axons, dendritic pruning, and myelination. It plays an important role in improving the connectivity between brain networks and improving signal transmission efficiency. The thinning of association cortices is seen as a reliable marker of maturity in healthy children (36, 37). The difference in cortical thickness between children with HIV and uninfected HEU children was predominantly in the association cortex with a few areas of excessive thinning, which may be the result of excessive synaptic pruning or abnormal plasticity. However, cross-sectional results are unavoidably affected by individual differences and groups. Our longitudinal study will overcome these limitations.

Neuroimaging has provided evidence of temporal hierarchy on typical brain development, with the primary cortices develop first and the association cortices continue to develop until the early three-decade of life. Sowell et al. mapped gray matter density in 176 typical development individuals aged 7 to 87 years and found that there was a significant non-linear decrease in gray matter density with age, especially on the lateral/dorsal frontal and parietal cortices. The primary sensorimotor regions, such as the visual, auditory, and the somatomotor cortices, begin to myelination at an early stage and exhibits a more linear developmental pattern, while the myelinization of the advanced functional cortex of the frontoparietal regions can continue into adulthood. In particular, the language cortex at the temporal cortex has the longest maturation process (16), and finally mature brain regions (frontal lobes) that involve advanced functions such as executive function and attention, but in this process not some brain regions are fully mature and other brain regions begin to develop (17). These studies indicate that the order of cortical maturation is consistent with the development of related cognitive abilities. Cortical thinning should occur first in the sensorimotor area, followed by the associated area, and finally in the more advanced cortical area. In our study, HIV+ children showed cortical maturation in multiple brain regions from primary to advanced functional regions after 1 year, but the brain regions involved were mostly sensory motor cortex and limbic systems. The range and extent of the HIV+ group's cortical maturation are significantly lower than that in HEU children. This pattern of high-function-associated cortical lagging development (especially in the left parietal lobe and bilateral occipital lobes) may be the basis for the manifestation of neurocognitive abnormalities and atypical brain development.

Cortical morphometry of neuroimaging has revealed atypical development in several brain diseases including schizophrenia and hyperactivity disorder, etc. (38). In a longitudinal study of children with schizophrenia, the developmental pattern of temporally and spatially thinning of the cerebral cortex was shown and was first detected in the parietal cortex associated with visual space and associative thinking. And this pattern of gray matter loss is related to the severity of mental symptoms (39). However, Shaw et al. showed that 223 children with attention-deficit/hyperactivity disorder (ADHD) showed that the age at which the peak cortical thickness was reached significantly later than that of normal children, especially in the prefrontal cortex. It indicates that there is a significant development delay in cortical maturation in children with ADHD (40). The different results of these studies suggest that changes in the basic maturation model of the cerebral cortex may be the basis of neurodevelopmental disorders. This process, together with social psychology and environmental factors, affects the development of individual cognitive abilities. The independent and interactive effects of these various factors may play an important role in brain structural changes and HIV-associated neurocognitive disorders in HIV+ patients. In the future, more in-depth clinical and basic research is needed to determine the pathophysiological mechanisms and potential therapeutic targets (41, 42).

Finally, we need to emphasize the role of white matter damage in the morphometric changes of HIV+. These cortical morphological changes found in this study may be partly due to the contribution of white matter damage. The axonal and myelin development of white matter has an important influence on the coordinated development of the gray matter. Recently, several studies have also pointed out that damage of the white matter structural connectivity that link the frontal, temporal, and occipital regions, as we reported, constitutes the basis of damage in HIV transmitted infection children, which suggests that the white matter damage may also be the basis for abnormal development of gray matter morphology in these population, but the relationship between them is still an open question, both for the typical development and HIV infected children. This relationship can be further analyzed and explored in future research (12, 43–45).

## Limitations

There are several limitations in this study. Firstly, because the data of this type of population is extremely rare, the sample size is very small, even if this study is obtained by using an unique opportunity. Secondly, the results reported in this paper only involved 1-year follow-up, and further follow-up is needed to obtain a detailed description of their developmental trajectories, and relevant research is continuing. In addition, because the subjects in this study were from different regions of the country, it is not very easy to completely and accurately obtain their previous complications.

## Conclusion

This cross-sectional and longitudinal study found that HIV-infected pubertal children showed a delayed cortical maturation (mainly in the left parietal lobe and bilateral occipital lobe) with atrophy (mainly occurs in the right frontal lobe, parietal lobe of the higher functional areas and multiple motor areas). This abnormal pattern of cortical development may be the structural basis for cognitive impairment in HIV+ children. Longer-term longitudinal studies are needed in the future to verify and improve this conclusion.

## Ethics Statement

The study was approved by the Medical Ethics Committee of Zhongnan Hospital of Wuhan University, and a written and informed consent was made from all participants or their guardians in accordance with the Helsinki Declaration of 1975 (and as revised in 1983), following a complete description of the measurements. These methods were carried out in accordance with the approved guidelines and regulations.

## Author Contributions

XY designed this study and wrote the paper, LG analyzed data, and contributed to writing of the paper. XY, HW, ZY, JF, JC, and QL performed experiments together. HX and XG guided the entire experimental process.

### Conflict of Interest Statement

The authors declare that the research was conducted in the absence of any commercial or financial relationships that could be construed as a potential conflict of interest.

## Abbreviations

AIDS: acquired immune deficiency syndrome
HIV: human immunodeficiency virus
HIV+: HIV-infected
MRI: magnetic resonance imaging
GMV: gray matter volume
HEU: HIV-exposed uninfected
VBM: voxel-based morphometric
CT: cortical thickness
MNI: Montreal neurological institute
FWE: Family Wise Error
TFCE: threshold-free cluster enhancement
OFC: orbital frontal cortex.
